# Recent Advances in Electrochemiluminescence and Chemiluminescence of Metal Nanoclusters

**DOI:** 10.3390/molecules25215208

**Published:** 2020-11-09

**Authors:** Shuang Han, Yuhui Zhao, Zhichao Zhang, Guobao Xu

**Affiliations:** 1 School of Science, Shenyang University of Chemical Technology, Shenyang 110142, China; unihanshuang@163.com (S.H.); Zhaoyuhui@ciac.ac.cn (Y.Z.); 2 State Key Laboratory of Electroanalytical Chemistry, Changchun Institute of Applied Chemistry, Chinese Academy of Sciences, Changchun 130022, China; 3 University of Science and Technology of China, Hefei 230026, China

**Keywords:** nanoclusters, luminophore, catalysts, quenchers, chemiluminescence, electro- chemiluminescence, fluorescence, aggregation-induced emission, sensors

## Abstract

Metal nanoclusters (NCs), including Au, Ag, Cu, Pt, Ni and alloy NCs, have become more and more popular sensor probes with good solubility, biocompatibility, size-dependent luminescence and catalysis. The development of electrochemiluminescent (ECL) and chemiluminescent (CL) analytical methods based on various metal NCs have become research hotspots. To improve ECL and CL performances, many strategies are proposed, from metal core to ligand, from intermolecular electron transfer to intramolecular electron transfer. Combined with a variety of amplification technology, i.e., nanostructure-based enhancement and biological signal amplification, highly sensitive ECL and CL analytical methods are developed. We have summarized the research progresses since 2016. Also, we discuss the current challenges and perspectives on the development of this area.

## 1. Introduction

Metal nanoclusters (NCs), comprising only a few to roughly hundreds of metal atoms, have a metal core-protective agent shell structure. Owing to the size of metal NCs approaching the Fermi wavelength of electrons, the spatial confinement of free electrons in metal NCs generates discrete electronic transitions, thereby exhibiting intriguing molecular-like properties. Therefore, metal NCs are deemed to bridge the gap between molecules and nanoparticles. In recent years, they have attracted a great deal of research in the applications of cellular imaging and chemical/biological detection owing to small size, excellent biocompatibility, good stability, distinctive catalytical activity, optical and electrochemical properties [1,2,3,4,5,6,7].

ECL phenomenon of fluorescent Ag NCs was studied for the first time in 2009 [8]. In 2011, Chen’s group and Zhu’s group found that Au NCs/triethylamine (TEA) system and Au NCs/potassium persulfate system had ECL phenomenon, respectively [9,10,11]. Based on these, they established new methods for the determination of Pb^2+^, dopamine and hydrogen peroxide. Subsequently, Yuan et al. realized the highly sensitive detection of phenols, microRNAs and dopamine with Au, Ag, and Cu NCs as ECL probes and potassium persulfate or hydrazine as coreactants [12,13,14]. Up to now, metal NCs, such as Au, Ag, Cu, Pt, Ni and alloy NCs have been widely studied as luminophores, catalysts, or quenchers in ECL system. Among them, there are many studies on luminophores. Unfortunately, the ECL efficiency of metal NCs is usually low, which significantly limits their sensing capability and application. To solve this problem, many strategies, such as valence state regulation [15,16], peroxidation [17], host-guest recognition [18], tuning ligand effects [19], covalent bonding of coreactants [20], have been reported recently. Many reviews have reported the ECL of metal NCs [21,22,23,24,25,26], but there is no systematic review on the methods to improve the luminescent efficiency of metal NCs.

Compared with ECL, metal NCs have been considered in CL applications since 2011 [27]. The CL signal of conventional molecular systems is often weak due to low quantum efficiency. Metal NCs could possess catalytic activity [28,29]. Therefore, the catalytic activity of metal NCs in CL system has been studied widely. In order to gain better analytical performance, some valuable researches have been done to improve catalytic activity of metal NCs, i.e., alloy NCs, synergistic effect of carbon nanomaterials and so on [30,31,32]. However, there are few studies on metal NCs as luminophores or quenchers [33,34,35,36,37].

In some aspects, fluorescence, CL and ECL of metal NCs seem to be interlinked or related. For example, the alloy NCs can improve the catalytic efficiency of CL and the luminescent efficiency of ECL [30,38,39,40,41]. Meanwhile, the aggregation-induced emission (AIE) is an effective strategy to enhance the fluorescence of metal NCs [2,42]. The implementation of the AIE strategy to the ECL field of metal NCs have been done to design metal NCs for high ECL efficiency [43,44].

The synthesis, ECL and CL applications of metal NCs before 2016 have been reviewed by us [45]. Thus, the purpose of this review is to summarize and highlight the recent five years works on metal NCs used in ECL and CL with a focus on the roles of metal NCs, methods or strategies for enhancing luminescence or catalytic properties of metal NCs and their analytical applications.

## 2. Electrochemiluminescence of Metal NCs

Since the ECL property of fluorescent Ag NCs was reported in 2009 for the first time [8], both anodic and cathodic ECL applications of metal NCs have been widely studied. These researches revealed versatile metal NCs, such as luminophores, catalysts and quenchers, and provided more possibility in the discovery of novel highly efficient ECL systems.

### 2.1. Metal NCs as ECL Luminophores

Au NCs have become the most investigated metal NCs in ECL applications. Studies of the ECL performance of other single metal NCs and alloy NCs are still in the preliminary stages and success in raising ECL efficiency has been limited. The common synthesis strategies of metal NCs are to choose appropriate templates and reducing reagent or templates with reductive properties, such as lipoic acid (LA), bovine serum albumin (BSA), methionine, glutathione (GSH), *N*-acetyl-L-cysteine, and DNA, to form metal NCs. However, the relatively low ECL efficiency of metal NCs has restricted their further applications. Another hurdle to the improvement of ECL efficiency is the unclear mechanism of the ECL, which prevents the innovation of new metal NCs.

**Figure 1:**
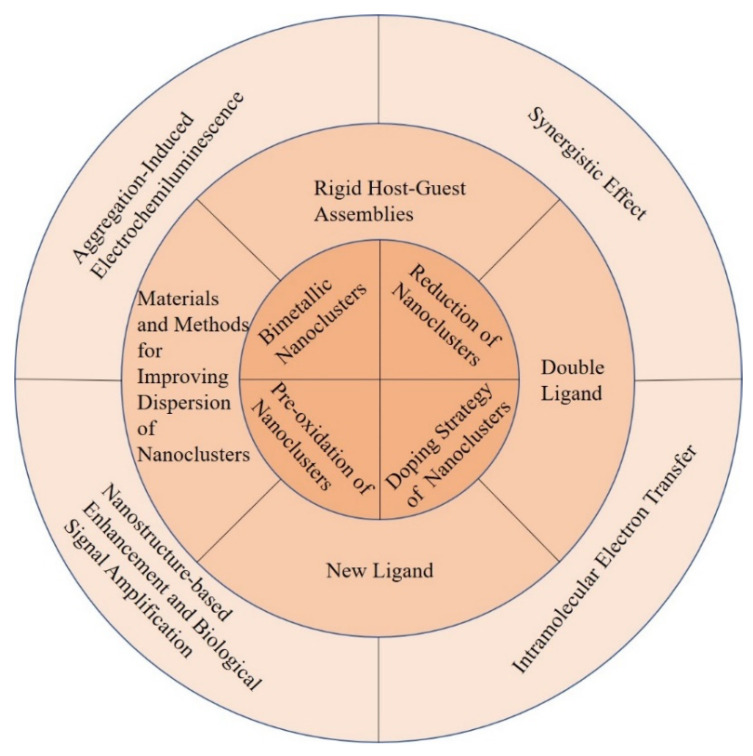
Schematic representation of the strategies for improving the ECL efficiency of metal NCs.

To date, a large number of efforts have been made to improve the ECL efficiency of metal NCs. Many studies are illustrated from the following perspectives: metal core valence states of metal NCs, bimetallic core, single metal core doped with electron rich rare earth elements, the number and category of template, coreactant, coreaction accelerator, synergistic effect and plasmon resonance enhancement effect of nanomaterials, nanostructure substrates, and bio-signal amplification effect (Figure 1). In addition, the researchers realized the intramolecular electron transfer by covalently linking luminophores and coreactants or luminophores, coreactants and coreaction accelerators, which differed from the original inefficient intermolecular electron transfer. Furthermore, a lot of researches have adopted a combination of two or three methods instead of a single approach to improve ECL efficiency, for example the combination of the intramolecular electron transfer and target-catalyzed hairpin hybridization amplification strategy [46].

#### 2.1.1. Au NCs as ECL Luminophores

Au NCs with good water solubility, excellent stability, ease of synthesis and modification have attracted wide interest in ECL field from the fundamental to the analytical applications [47,48]. They have been shown to be prospective nanoscale ECL luminophores that are being extensively explored [49]. However, the relatively low ECL efficiency of Au NCs has been a main obstacle for their better applications. Many efforts from internal metal core to external environment, from intermolecular to intramolecular electron transfer have been made to improve the ECL efficiency.

One important parameter of highly luminescent metal NCs is the valence state of metal atoms in NCs. The reduction degree of Au NCs is positively related to the enhancement of the ECL signals of Au NCs. Peng et al. investigated the valence state effect of Au on the ECL performance of *N*-acetyl-L-cysteine stabilized Au NCs (NAC/Au NCs) [15]. Once the NAC/Au NCs was reduced by either electrochemical or chemical method, an enhanced ECL was observed. NAC/Au NCs have different valence states of Au, typically as Au(I) and Au(0). Au(I) is responsible for the fluorescence of NAC/Au NCs and Au(0) core is main contribution of NAC/Au NCs in the ECL. Thus, the enhanced ECL signal of electro-reduced NAC/Au NCs was about 30 times higher than that of NAC/Au NCs with K_2_S_2_O_8_ as the coreactant. The ECL quantum efficiency of electro-reduced NAC/Au NCs was 4.11% with [Ru(bpy)_3_]^2+^ as a reference system. This approach had been successfully extended to other Au NCs, such as GSH stabilized Au NCs (GSH/Au NCs). Both the electrochemical and chemical reduction of the GSH/Au NCs showed strong and efficient ECL signals. Therefore, this study presents a new idea to design ECL device from other functional-metal based NCs, but also extends the huge potential application in the ECL sensing. Based on the excellent quenching effect of GSH on the ECL of the NAC/Au NCs, the same group constructed a very simple NAC/Au NCs-based ECL nanosensor for the detection of GSH with a low detection limit of 3.2 × 10^−10^ M [50]. More interestingly, the oxidation state of the Au NCs also played a critical role in the ECL. The same group reported a record high ECL quantum yield (*Φ*_ECL_) of 66% achieved by pre-electrooxidation of L-methionine stabilized Au NCs (Met/Au NCs) with TEA as a coreactant [17]. It was suspected that the electrooxidized product of Met/Au NCs (Met/Au NCs ^•+^) could be accumulated on the electrode surface to enhance the *Φ*_ECL_. This method can be successfully extended to other Au NCs, such as dithiothreitol (DTT) stabilized Au NCs (DTT/Au NCs) and BSA/Au NCs. The ECL signals of pre-oxidized DTT/Au NCs and pre-oxidized BSA/Au NCs enhanced by 20.3-fold and 5-fold, respectively. BSA is the largest ligand and the electron transfer rate is slowest. Thus, pre-oxidation is more effective for ligand shells, facilitating the electron transfer. This work is an important breakthrough in the ECL field, which not only provides a new method for in-depth study of ECL but also opens up a new avenue for designing and developing high performance ECL probes.

Besides the ECL intensity, the valence states of Au NCs could also affect the ECL wavelength. Kim et al. found that the sources of the ECL and photoluminescent (PL) emissions of the Au NCs were different and the ECL of Au NCs was closely related to the presence of Au(0) in the NCs [51]. Water-soluble GSH/Au NCs reduced by NaBH_4_ with TEA as coreactant can generate the unusual near-infrared (NIR) ECL. The research showed that the NIR ECL at 800 nm was ascribed to the Au(0)–GSH motif in the GSH/Au NCs in water, while the PL at 610 nm was attributable to the Au(I)–GSH motif on the surface of GSH/Au NCs. However, Au NCs synthesized by chemical method are usually polydisperse. Most of the studies on the ECL of Au NCs are based on polydisperse Au NCs. In contrast, the synthesis of monodispersed Au NCs of high ECL remains a challenge. The same group further studied the ECL of individual GSH/Au NCs fractionated by gel electrophoresis with TEA as the coreactant [52]. The individual Au NCs possessed unique and significant differences in ECL. When the ratio of Au(I)/Au(0) in the individual Au NCs decreased, NIR ECL of GSH/Au NCs became dominant. This study suggests that the oxidation states of the individual Au NCs will impose significant effect on the ECL wavelength of the clusters. Therefore, the present study can provide a deep insight into the origin of NIR ECL of water-soluble GSH/Au NCs. The findings of Kim and Peng demonstrated that the valence states of Au NCs could both affect the ECL wavelength and intensity of Au NCs.

Furthermore, the change of Au core composition could also affect the ECL signal of Au NCs by the synergistic effect of the two kinds of atoms in NCs core. For example, one study has demonstrated that the doping of Ag into BSA/Au NCs with the molar ratios of 6:1 (Au:Ag) led to the 5-fold ECL emission compared to the individual BSA/Au NCs [38]. A Hg^2+^ ECL sensor was developed based on Hg^2+^-induced ECL quenching of BSA/AuAg NCs using TEA as coreactant through the formation of strong metallophilic bonding (Au-Hg^2+^/Ag-Hg^2+^). The detection limit of Hg^2+^ was 5 nM lower than the standard of the World Health Organization for Hg^2+^ in drinkable water (30 nM). A similar study also suggested that the ECL signal of GSH stabilized AuAg NCs (GSH/AuAg NCs) was about 100 times higher than that of GSH/Au NCs [40]. The rigid structure of metal core by doping another metal atom could reduce nonradiative transition and thus enhance luminescence of ECL. Chen et al. reported the NIR ECL of rod-shape bimetallic Au_12_Ag_13_ NCs [41]. With Ru(bpy)_3_^2+^ as reference, the self-annihilation ECL of the Au_12_Ag_13_ NCs was about 10 times higher. The coreactant ECL of Au_12_Ag_13_ NCs was about 400 times stronger than that of Ru(bpy)_3_^2+^/tripropylamine (TPrA) system. The origin of the strong ECL of Au_12_Ag_13_ was attributed to the 13th Ag atom at the central position which stabilized the charges on the LUMO orbital and made the rod-shape Au_12_Ag_13_ core more rigid. The study could guide the design and syntheses of other NCs or materials in general to achieve improved properties and further affirm the structure-function correlations.

**Figure 2:**
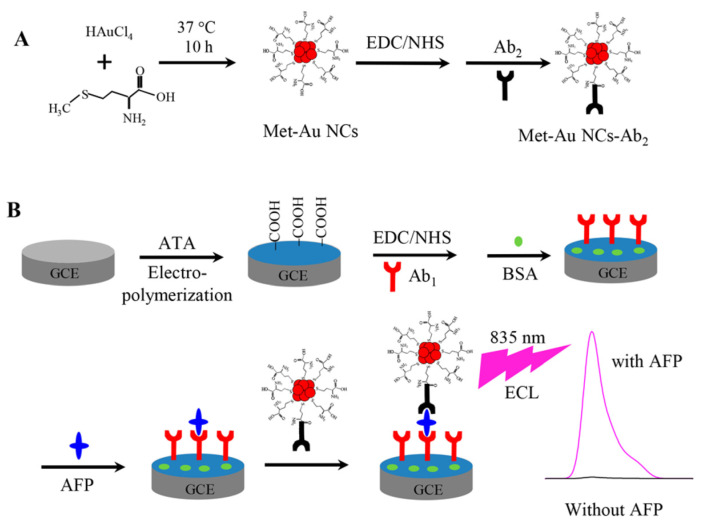
Schematic Illustration of the proposed NIR ECL sensing strategy with Met/Au NCs as ECL tags. (**A**) Synthesis of Met-Au NCs-Ab_2_. (**B**) Immobilization of Met-Au NCs-Ab_2_ onto the GCE surface via the proposed sandwich immunoassay strategy. Reprinted from [53] with permission from ACS.

The surface ligand of Au NCs plays an important role in ECL reaction. The study found that the ECL intensity of Met/Au NCs was about five times higher than that of BSA/Au NCs with K_2_S_2_O_8_ as the coreactant [19]. The oxidized dopamine can lead to the ECL quenching of the Met/Au NCs. The method was used to the detection of dopamine released by cells. The Met/Au NCs showed yellow color luminescence and BSA/Au NCs showed red color luminescence. Therefore, Au NCs-based ECL probes with tunable luminescent properties have potential applications in bioanalysis such as multi-labeling techniques. Yu et al. used the same Met and the similar synthetic method to synthesize Met/Au NCs which demonstrated NIR ECL emission of 835 nm with triethanolamine as coreactant and a 75 times enhanced ECL compared with BSA/Au NCs [53]. As shown in Figure 2, a sandwich-type NIR ECL immunosensor was constructed with the Met/Au NCs as tags and α-fetoprotein (AFP) as a model analyte and exhibited a wide linearity range from 3 fg·mL^−1^ to 0.1 ng·mL^−1^ with a detection limit of 1 fg·mL^−1^.

The ECL enhancement effect of two ligands is better than that of one ligand. Wang’s group proposed a novel anodic ECL emitter of thioglycol/glutathione dual ligand-coated Au NCs (TG/GSH/Au NCs) [54]. TG/GSH/Au NCs showed a new ECL peak at a lower potential of + 1.4 V with TEA as coreactant and about 5 times enhanced ECL than that of GSH/Au NCs. Other common thiol compounds had no similar effect.

Inspired by the enhanced PL of Au NCs with a rigid shell, forming rigid host-guest assemblies around Au NCs by introducing another molecule could reduce the non-radiative relaxation of the excited states in ECL processes to enhance the ECL performance of Au NCs. Yang et al. mixed L-arginine (ARG) and 6-aza-2-thiothymine (ATT) protected Au NCs (ATT/Au NCs) to form the rigid host-guest assemblies ARG/ATT/Au NCs via hydrogen bonding [18]. The rigid shell around Au NCs core enhances the radiative-charge-transfer within the Au NCs whilst keeps their excited states unchanged. Thus, the ECL of ARG/ATT/Au NCs was enhanced dramatically than ATT/Au NCs without coreactant and was about 70 times higher to that of ATT/Au NCs with TPrA as coreactant. This study presents a new method for screening novel ECL luminophores with enhanced performance.

The ECL intensity can also be enhanced by using novel coreactants. Wang’s group reported the adoption of chelating agent EDTA and commonly used buffer 4-(2-hydroxyethyl)-1- piperazineethanesulfonic acid (HEPES) as coreactants for the enhancement of NIR ECL from LA-stabilized Au NCs (LA/Au NCs) at physiological pH [55,56]. Metal ions can modulate ECL signal effectively. Mg^2+^ had profound impacts on the ECL features of LA/Au NCs-EDTA system through the complexation with EDTA and the interaction with LA/Au NCs. Zn^2+^ was found to enhance the ECL of LA/Au NCs-HEPES system while Mg^2+^ and Ca^2+^ lowered the ECL signal. The ECL intensity of LA/Au NCs system displayed optimal responses at physiological pH which made HEPES better suited over EDTA for the corresponding applications. The work applies to other ions and other amine-based good’s buffers.

The poor conductivity of protein coated on the surface of metal NCs could limit the mass transport and electron transfer, lower the ECL efficiency of NCs and restrict their applications [57]. Coreaction accelerator including some nanoparticle or active micromolecule can improve the ECL emission of NCs through effectively reducing the coreactant to a stronger oxidative or reductive intermediate radical. Jia et al. utilized highly-branched Cu_2_O as the coreaction accelerator in BSA/AuNCs-K_2_S_2_O_8_ ECL system [58]. Highly-branched Cu_2_O can catalyze K_2_S_2_O_8_ to produce more radical anion SO_4_^•−^, which can oxidize Au NCs^•−^ to generate more Au NCs*, thus doubling the ECL intensity. The same group also synthesized Cu_2_S snowflake as the coreaction accelerator to produce more cationic radicals TEA^•+^ which could improve the ECL intensity of BSA/Au NCs-TEA system [59]. The biosensor exhibited ultrahigh immune recognition to procalcitonin with the detection limit as low as 2.36 fg mL^−1^. Coreactant accelerator can be also generated in situ. Zhang et al. developed a highly sensitive ECL method for detection of acetylthiocholine (ATCI) based on the catalysis of acetylcholinesterase (AChE) and BSA/AuNCs-K_2_S_2_O_8_ ECL system [60]. AChE catalyzed ATCI to produce thiocholine, which acted as the coreactant accelerator to improve the ECL emission. The strategy obtained a low detection limit of 0.17 nM for ATCI.

However, the long electron transfer path and great energy loss in the intermolecular ECL reaction between luminophores and coreactants restrict its ECL efficiency [57]. Binary or ternary nanostructure formed by luminophores, coreactants and coreaction accelerators via covalent attachment is benefit for highly efficient ECL [20,46,57,61]. Wang et al. combined LA/Au NCs as the luminophore and *N*,*N*-diethylethylenediamine (DEDA) as the coreactant to form the binary nanostructure [20]. The NIR ECL signal of binary nanostructure was about 17 times higher than that from Ru(bpy)_3_^2+^/TPrA system. The design reduced the complication of mass transport between the reactants during the lifetime of radical intermediates involved in conventional ECL generation pathway. The intracluster reactions were highly advantageous for applications by eliminating additional and high excess coreactants otherwise needed. The study opened a new avenue for the designment of high-efficiency Au NCs ECL system. Combining this type of Au NCs ECL system with DNA amplification technology, ECL ultrasensitive detection can be realized. Yang et al. designed a high efficiency ECL label of binary nanostructure with thioctic acid capped Au NCs (TA/Au NCs) as the luminophore and *N*,*N*-diisopropylethylenediamine (DPEA) as the coreactant for the ultrasensitive detection of mucin 1 (MUC1) by employing multi-site landing DNA walker as signal amplification strategy [61]. The ECL aptasensor of MUC1 was established with a detection limit of 0.54 fg mL^−1^. The study provided a new method for the development of metal NCs-based ultrasensitive ECL platform for biomolecule detection in clinical analysis.

**Figure 3:**
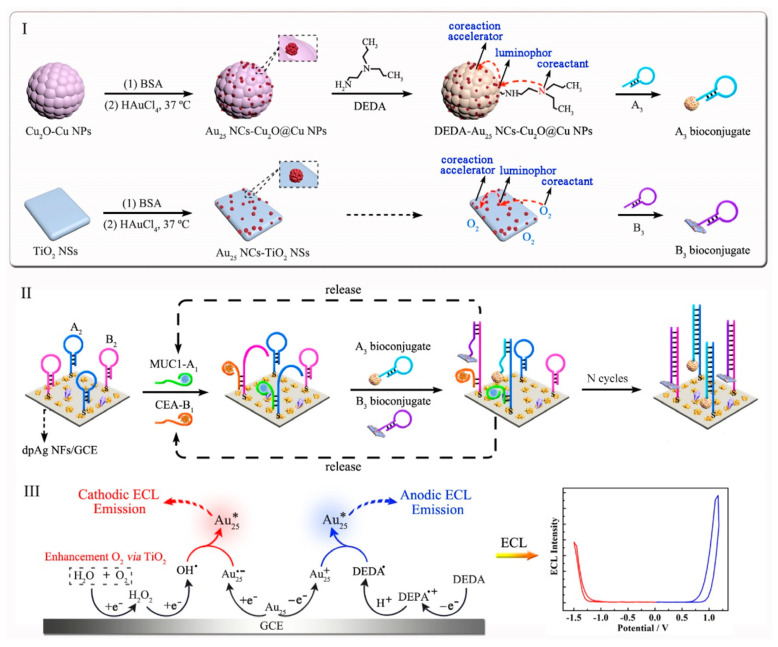
Schematic diagram showing fabrication of the ECL aptasensor. (**I**) synthesis of the A_3_ bioconjugate (A_3_/Au_25_ NCs-Cu_2_O@CuNPs-DEDA) and the B_3_ bioconjugate (B_3_/Au_25_ NCs-TiO_2_ NSs), (**II**) working principle of the aptasensor for MUC1 and CEA simultaneous detection, and (**III**) a possible ECL mechanism of simultaneous cathodic and anodic ECL emissions of the Au_25_ NCs on an interface. Reprinted from [46] with permission from ACS.

In order to further improve ECL efficiency of metal NCs, a highly efficient ECL label containing luminophore, coreactant and coreaction accelerator was designed in a nanostructure [57]. BSA/Au NCs as the luminophore, tris(3-aminoethyl)amine (TAEA) as the coreactant and Pd@CuO nanomaterial as the coreaction accelerator via covalent attachment formed the BSA/Au NCs-TEAE-Pd@CuO ternary nanostructure as highly efficient ECL label for constructing an ultrasensitive biosensor of carcinoembryonic antigen (CEA). The ternary ECL nanostructure showed the maximum ECL intensity and the lowest luminous potential. This indicated that an intramolecular ternary system with faster electronic transfer and more effective energy transmission exhibited optimal ECL performance as compared with others ECL systems. The principle was that both Au NCs and TAEA in nanostructure were oxidized to form Au NCs^+^-TAEA^•+^. Furthermore, the tertiary amine of TAEA lost a proton to generate Au NCs^+^-TAEA^•^ intermediate. When Pd@CuO as the coreaction accelerator promoted intramolecular electron transfer and energy transmission, more active radical Au NCs^+^-TAEA^•^ produced the excited state Au NCs*-TAEA, which returned to the ground state and emitted intense light. The detection limit of this method was as low as 16 fg mL^−1^ which was comparable to other CEA detection literatures. Using the similar design strategy, the same group developed a ECL method for simultaneous detection of CEA and MUC1 based on the BSA/Au NCs with different coreactants and coreaction accelerators for the first time [46]. BSA/Au NCs was chosen as the only luminophore owing to its simultaneous cathodic and anodic ECL emissions under continuous cathodic and anodic scanning. As shown in Figure 3, Au NCs-TiO_2_ nanosheets as the cathodic ECL probe exhibited outstanding cathodic ECL signal by combining TiO_2_ nanosheets as the coreaction accelerator with O_2_ as the coreactant. The ternary nanostructure of BSA/Au NCs-Cu_2_O@Cu nanoparticles-*N*,*N*-diethylethylenediamine (BSA/Au NCs-Cu_2_O@Cu NPs-DEDA) acted as the anodic ECL probe to obtain excellent anodic ECL emission. On the basis of cathodic and anodic Au NCs nanocomposites as high-efficient ECL probes and target-catalyzed hairpin hybridization as signal amplification strategy, an ultrasensitive ECL aptasensor was constructed for simultaneous detection of CEA and MUC1, with detection limits of 5.8 fg mL^−1^ and 0.43 pg mL^−1^, respectively. The proposed strategy easily solved one main technical challenge of cross reactions of dual-luminophores for dual-biomarker detection and realized single-step detection of dual biomarkers simultaneously, which initiated a new thought in realizing a new generation of dual-biomarker ECL detection beyond the traditional ones in sensing analysis and diagnostic sensing.

Guided by the design principles toward AIE, the AIE characteristics of the metal NCs were found [43,44]. Drying 6-aza-2-thiothymine (ATT) protected Au NCs (ATT/Au NCs) on glass carbon electrodes enhanced emission by aggregation-induced ECL (AIECL) with TEA as the coreactant [43]. The quantum yield of ATT/Au NCs in TEA was 0.8%, while dried ATT/Au NCs was 78% with Ru(bpy)_3_^2+^ as a reference. It was speculated that drying process effectively suppresses the vibration and rotation of ATT and thus the nonradiative relaxation. Thus, the ECL quantum yield of ATT/Au NCs was enhanced significantly. Besides drying, aggregation of Au NCs caused by metal ions can also improve ECL efficiency. Wang et al. investigated the anodic ECL features of adenosine triphosphate (ATP) protected Au NCs (ATP/Au NCs) before and after Ca^2+^ induced aggregation [44]. The ECL of ATP/Au NCs increases significantly 50-fold after adding Ca^2+^. TEM clearly showed the aggregated rod-like ATP/Au NCs assembly caused by the strong binding of Ca^2+^ to phosphate group on the ATP. It was indicated that the AIECL behaviors occurred. A calmodulin ECL sensor was developed based on the binding of Ca^2+^ to calmodulin with a limit of detection 0.1 μg mL^−1^. Other strategies of nanostructure substrate amplification effect, biological amplification effect, ECL resonance energy transfer and synergistic effect are also used to improve the ECL performances of Au NCs [62,63,64].

#### 2.1.2. Other Monometallic NCs (Ag, Cu, Pt, Ni) as ECL Luminophores

Though Au NCs are observed to show high ECL intensity commonly used in ECL systems, the ECL luminescent properties of other metal NCs have been gradually explored, such as Ag, Cu, Pt and Ni NCs. At present, the methods to improve the ECL luminescence intensity of Ag NCs are to design DNA structure, improve the stability and uniformity of silver nanoclusters synthesis, and enhance the ECL signal; or to combine nucleic acid amplification technology, co-reaction accelerator or surface plasmon resonance (SPR) of metal nanoparticles to increase ECL.

DNA is the most commonly used template for Ag NCs synthesis. The ECL properties of Ag NCs are highly dependent on the nature of the template molecules. Resolving their stability and homogenous questions are quite attractive for the enhancement of ECL efficiency of Ag NCs. Feng’s team used a triplex DNA template for the synthesis of site-specific, homogeneous and highly stable Ag NCs [65]. Ag NCs were formed in the very position of CG.C^+^ triplet site in triplex DNA. As the number of CG.C^+^ was more, the ECL intensity of Ag NCs was stronger. A strong ECL signal was observed for the triplex DNA-Ag NCs solution, while comparable weak ECL was observed when duplex DNA and single strand DNA used as templates. A signal-on pattern of ECL sensor was developed for the detection of biothiols based on the enhanced catalytic reaction and a robust interaction between the triplex-AgNCs and cysteine, by influencing the microenvironment provided by DNA template. The triplex-templated Ag NCs would be promising candidates in the ECL analysis with the good stability and easier preparation.

**Figure 4:**
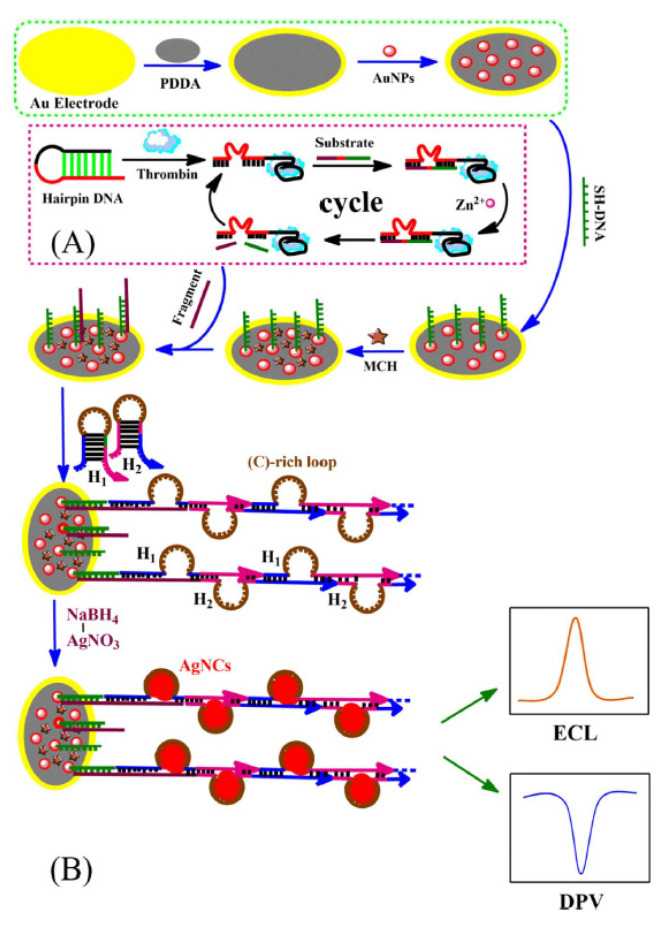
Schematic representation versatile ECL and electrochemical detection of thrombin based on silver nanoclusters in situ synthesized by multiple signal amplification strategy. (**A**) DNAzyme-assisted amplification, and (**B**) HCR process. Reprinted from [66] with permission from Elsevier.

The combination of ECL of Ag NCs with the nucleic acid based biological amplification strategy is another common ECL enhancement method. Jie’s group developed a highly sensitive Ag NCs for thrombin detection based on DNAzyme-assisted target recycling and hybridization chain reaction (HCR) multiple amplification strategy [66]. As shown in Figure 4, thrombin recognition opened the hairpin DNA and initiated the binding of the substrate with the DNAzyme (red line). The fragment of single-stranded DNA (ssDNA) was released followed by hydrolytic cleavage with catalytic effect of Zn^2+^. This induced the DNAzyme recycling and generation of a large amount fragment of ssDNA as target DNA to execute more reactions. The introduced H1 and H2 can trigger the HCR process to form linear dsDNA with cytosine-rich templates, which facilitated the in-situ syntheses of Ag NCs as ECL and electrochemical tags. The detection limit of thrombin was as low as 0.1 fM, showing potential of the method for other biomolecules detection.

In addition to nucleic acid signal amplification, it can also be combined with coreaction accelerators to enhance ECL. Using in situ formed Ag NCs and Fe_3_O_4_-CeO_2_ as coreaction accelerator, Zhou et al. fabricated an ultrasensitive ECL biosensor for the detection of cyclin-D1 (CCND1) [67]. The ECL luminous efficiency of the Ag NCs on the electrode is significantly enhanced by the Fe_3_O_4_-CeO_2_ nanocomposites which promote the reduction of S_2_O_8_^2−^ to generate the strong oxidizing intermediate radical SO_4_^•−^.

A novel mechanism named surface plasmon-enhanced ECL was found to drastically enhance the ECL of Ag NCs. Wang et al. fabricated a high sensitivity and selectivity miRNA-21 biosensor based on surface plasmon-enhanced ECL with DNA templated Ag NCs as ECL luminophores and Au nanoparticles (Au NPs) as the localized surface plasmon resonance source [68]. The ECL enhancement of Ag NCs by Au NPs is related with the separation distance between Ag NCs and Au NPs and the electrodeposition time of Au NPs. Combined a cyclic amplification process with enzyme-free catalytic hairpin DNA, miRNA-21 was successfully detected with a wide linear range from 1 aM to 10^4^ fM and a relatively low detection limit of 0.96 aM.

**Figure 5:**
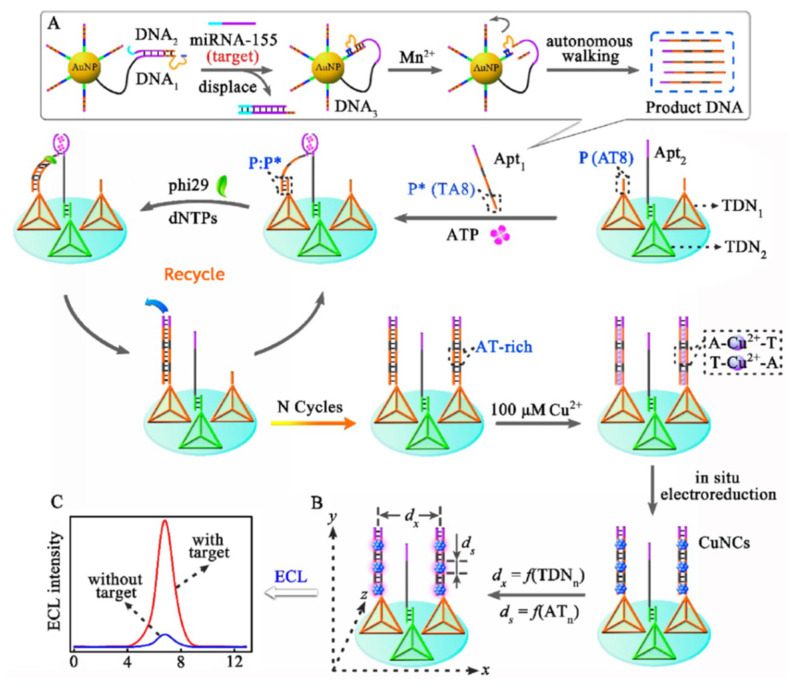
Schematic Illustration of the Nanocranes-Based Biosensor: (**A**) Target-Nucleotide Transduction-Amplification Strategy; (**B**) Programmable Modulation of the ECL Efficiency of Cu NCs; (**C**) Signal Comparison of miRNA-155 Detection (dx = Lateral Spacing of Cu NCs; ds = Particle Size of Cu NCs). Reprinted from [71] with permission from ACS.

Considering the high consumption of noble gold and silver, Cu NCs as a nontoxic and low-cost candidate, have gained growing interest in ECL applications. The anodic and blue ECL of the Cu NCs with hydrazine as coreactant for the determination of dopamine was first demonstrated by Yuan’s group [14]. However, the electron-transfer path between the Cu NCs in aqueous and the sensing interface was so long that much energy was lost and the ECL luminous of Cu NCs was inhibited. In addition, the tight molecular arrangement of Cu NCs stabilized by BSA would increase the inner-filter effect and impede the electrochemical activation of the internal ECL emitters. Therefore, the sequence-dependent synthesis of Cu NCs via the stable A-Cu^2+^-T bond was developed [69]. The longitudinal distribution of Cu NCs could be controlled onto DNA templates and the lateral distance could be designed by the DNA nanocrane. Based on this, the same group developed two kinds of highly sensitive Cu NCs for miRNA detection. One was based on the exonuclease (III-assisted amplification, HCR process and TiO_2_ as the coreaction accelerator [70]. The other one was based on the DNA nanocrane with binding-induced DNA assembly as manipulator and tetrahedral DNA nanostructure as base (Figure 5) [71]. This study had three novel ideas. First, the binding-induced DNA assembly finely manipulated the generation of AT-rich dsDNA. On the basis of the stable A-Cu^2+^-T, the DNA nanocranes realized a small quantity of miRNA-155 that triggered the generation of a large amount of Cu NCs. Second, TDN could not only improve the probe spacing to generate more Cu NCs but also lessen collision annihilation of excited-state species Cu NCs* for retaining remarkable ECL emission, which significantly promoted the sensitivity of the biosensor. Third, the reasonably designed biosensor simultaneously realized activity modulation of ECL probes (the DNA-stabilized Cu NCs) and target recycling amplification, achieving ultrasensitive analysis of miRNA-155 with the detection limit of 36 aM. With the ingenious combination of DNA nanocranes, the proposed biosensor exhibited excellent selectivity, accuracy, and stability and provided a dynamic sensing platform for various target analysis in diverse applications, such as early disease diagnosis and environmental analysis.

Recombination(composition) and doping are also the methods to improve the ECL efficiency of Cu NCs [72,73]. Liu et al. combined dithiothreitol functioned Cu NCs (DTT/CuNCs) and electrocatalyst material carbon nitride nanosheets (CNNSs) to form a nanocomposite material (DTT/Cu NCs/CNNSs) [72]. The pure CNNSs delivered high but unstable ECL intensity performance and the ECL intensity of DTT/Cu NCs was the weakest with K_2_S_2_O_8_ as coreactant. But the DTT/Cu NCs/CNNSs nanocomposite provided the excellent and reasonably stable ECL signal. Hg^2+^ can chelate the free SH groups on the surface of DTT/Cu NCs and suppress the formation of anion-radicals of DTT/Cu NCs/CNNSs. The method was successfully applied to the detection of Hg^2+^ in water pollution monitoring with a detection limit of 0.01 nM. Doping the rich electronic rare earth element into Cu NCs is another effectively method to improve the stability of Cu NCs and to increase ECL intensity. Zhuang et al. studied the ECL enhancement phenomenon of Eu^3+^ ion doped GSH stabilized Cu NCs (GSH/Cu NCs) for the first time [73]. Eu^3+^ ions can alter the surfaces of Cu NCs and drive the formation of new surface states (Eu(III) complex) to promote effective energy transfer from the host to the Eu^3+^ ions. The ECL intensity of Eu^3+^- GSH/Cu NCs increased by 3-fold and the stability of GSH/Cu NCs was enhanced greatly. The method provided a simple mode for turn-off detection of dopamine with a detection limit of 1.0 × 10^−11^ M. The presented results give us a clue to overcome the stability issues of metal NCs, provide a powerful tool for the development of ECL emitters, and may open up promising avenues to develop new ECL systems for medical and biological analysis.

Unusual Pt NCs and Ni NCs also exhibit ECL properties [74,75]. Graphene (GR) could immobilize Pt NCs and accelerate electron transfer between the electrode and coreactant TEA which enhanced the ECL intensity of GR/Pt NCs two times than that of Pt NCs [74]. Babamiri et al. developed a novel molecularly imprinted polymer (MIP)-ECL sensor for selective detection of creatinine based on BSA/Ni NCs as emitter and tri-*n*-propylamine as coreactant [75]. The ITO electrode was modified with a magnetic graphene oxide MIP film with BSA/Ni NCs-embedded in MIP. In the presence of creatinine, the ECL emission of BSA/Ni NCs was strongly quenched owing to the imprinted cavities of MIP film occupied.

### 2.2. Metal NCs as ECL Quenchers

**Figure 6:**
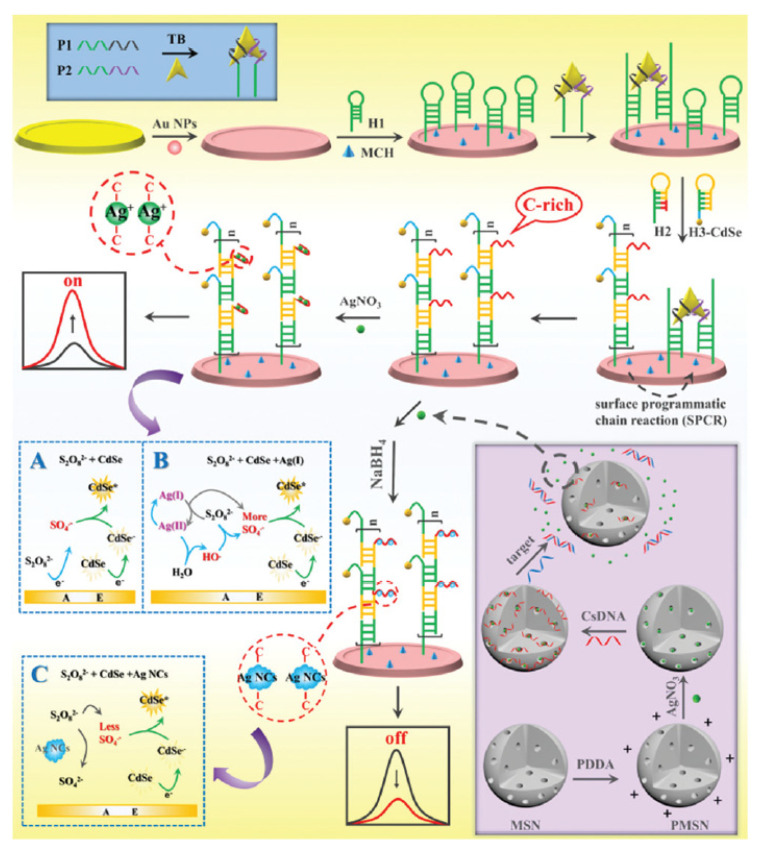
Schematic diagram of the “on–off” ECL biosensing platform for versatile detection of thrombin and miRNA-21 based on Ag(I) ion accelerated and Ag NC-quenched ECL combined with molecular machine-triggered chain reaction and MSN double amplification. (**A**) The ECL process of CdSe QDs + S_2_O_8_^2−^ system; (**B**) The ECL process of CdSe QDs + S_2_O_8_^2−^ system with Ag(I) ions as coreaction accelerators; (**C**) The ECL process of CdSe QDs + S_2_O_8_^2−^ system with Ag NCs as acceptors and CdSe QDs as donors. Reprinted from [76] with permission from RSC.

Generally, the luminous efficiency of quantum dots (QDs) is higher than that of metal NCs. When the absorption peak of the metal NCs matches the ECL emission peak of the QDs, ECL resonance energy transfer between the QDs and metal NCs can be performed. There are few reports on this aspect, some of which have been summarized in the previous review [45]. Recently, Jie et al. designed a versatile ECL method for detection of thrombin and microRNA-21 based on the Ag(I) ion-enhanced or Ag NCs quenched ECL of CdSe QDs [76]. Bipedal molecular machine (BMM)-triggered surface programmatic chain reaction (SPCR) coupled with mesoporous silica nanoparticle (MSN) multiple amplification is used to introduce plentiful QDs and Ag^+^ ions to significantly improve the ECL signal for sensitive ‘‘signal on’’ detection of thrombin. Due to the ECL resonance energy transfer (RET) between Ag NCs acceptors and CdSe QD donors, the ECL signal of CdSe QDs was quenched. In addition, Ag NCs competitively reacted with S_2_O_8_^2−^ to reduce the production of ECL co-reactive free radicals (SO_4_^−•^), which further decreased the ECL signal; thus a “signal off” ECL biosensor for ultrasensitive detection of microRNA-21 was developed (Figure 6). Yang et al. designed a wavelength-resolved ECL resonance energy transfer (ECL-RET) ratiometric immunosensor from Au NPs functionalized graphite-like carbon nitride nanosheets (Au-g-C_3_N_4_) to Au NCs [77]. The well-matched ECL emission spectrum of Au-g-C_3_N_4_ and absorption spectrum of Au NCs, as well as the absence of ECL emission of Au NCs, make the system an efficient and interference-free ECL-RET ratiometric system. As a consequence, a sensitive ECL biosensor for detection of cardiac troponin I was accomplished with a wide linear range from 50 fg mL^−1^ to 50 ng mL^−1^ and a low detection limit of 9.73 fg mL^−1^. This work enriched the wavelength-resolved ECL-RET system and provided an innovative reference for the development of more efficient and sensitive ECL-RET ratiometry.

### 2.3. Metal NCs as ECL Catalysts

Luo et al. proposed a sensitive ECL biosensor for detection of protein kinase activity (PKA) based on the BSA/Au NCs enhanced cathodic ECL of graphite-like carbon nitride material (g-C_3_N_4_)-S_2_O_8_^2−^ system [78]. After dropping the g-C_3_N_4_ onto the surface of a glassy carbon electrode (GCE), peptides were assembled by reaction with chitosan. Using PKA as a model kinase, BSA/Au NCs were immobilized onto the phosphorylated peptides modified g-C_3_N_4_/GCE by Au-S bond in the presence of adenosine 5′-[γ-thio] triphosphate (ATP-s) and PKA. The assembled BSA/Au NCs as the catalysts of the cathode ECL reaction of g-C_3_N_4_ can significantly enhance the ECL intensity. The resultant ECL signal of g-C_3_N_4_ was magnified 4.5 times compared with the absence of BSA/Au NCs. The proposed ECL platform is capable of analysing PKA activity of biological sample quantitatively and screening kinase inhibition qualitatively.

Pt NCs play double roles of the acceptor and the donor in the multiple ECL resonance energy transfer (RET) system [79]. The PL emission spectrum of the Alexafluor (AF) overlapped with both the UV-Vis absorption spectra of polyethyleneimine stabilized Pt NCs and tris(4,4′-dicarboxylic acid-2,2′-bipyridyl) ruthenium(II) dichloride (Ru(dcbpy)_3_^2+^). It is indicated that resonance energy transfer (RET) can be generated not only between AF and Pt NCs but also between AF and Ru(dcbpy)_3_^2+^. Then the PL emission spectrum of the Pt NCs overlaps with the UV-vis absorption spectrum of Ru(dcbpy)_3_^2+^, displaying the presence of RET between Pt NCs and Ru(dcbpy)_3_^2+^. Therefore Pt NCs can accept energy from alexafluor, transfer it to Ru(dcbpy)_3_^2+^ and then enhance ECL efficiency. The existence of multiple energy donor/acceptor pairs in the same nanostructure resulted in a shorter electron-transfer path, less energy loss and higher RET efficiency. Combined with target recycling amplification technology, the ECL efficiency is 1.78 times higher than the classic Ru(bpy) _3_^2+^. From what has been discussed above, a brief summary of the methods to improve ECL efficiency of metal NCs was shown in Table 1.

## 3. Chemiluminescence of Metal NCs

### 3.1. Metal NCs as CL Catalysts

The applications of metal NCs in CL mainly focus on the catalysis, and the research on metal NCs as luminophore and quencher is less. Metal NCs are served as catalysts based on the reaction between the metal NCs and the CL system including H_2_O_2_-nitrite [80], H_2_O_2_-fluorescein [81], luminol-H_2_O_2_ [82,83,84,85,86,87], KMnO_4_-rhodamine B [31,32], H_2_O_2_-rhodamine B [30], diperiodato- argentate-folic acid [88], KMnO_4_-rhodamine 6 G [89], H_2_O_2_-peroxymonocarbonate [90], luminol-NaIO_4_ [91], and K_3_Fe(CN)_6_-rhodamine 6 G [92]. In the presence of target, CL intensity is quenched or restored/enhanced. Up to now, there are about four ways to improve the catalytic activity of metal NCs in CL system: (1) charged metal NCs by modification; (2) bimetallic core NCs; (3) synergistic effect of nanomaterials; (4) Metal NCs encapsulated in uniform and well-ordered nano-porous structures. These strategies are also applied to improve the ECL efficiency of metal NCs.

#### 3.1.1. The Catalysis of Single Metal NCs

Chen et al. found that BSA/Au NCs can enhance the intensity of CL system, such as K_3_Fe(CN)_6_-rhodamine 6 G [92] and H_2_O_2_-fluorescein [81]. BSA/Au NCs can effectively catalyze the decomposition of H_2_O_2_ to produce double hydroxyl radicals. Hydroxyl radicals can react with HO_2_^−^ to generate O_2_^•−^ radicals. Rhodamine 6 G reacted with O_2_^•−^ radicals on the surface of Au NCs to generate the excited state intermediate more easily. Bisphenol A and catechol belong to polyhydroxy compounds. They have strong reducibility and can be used as scavenging agents of reactive oxygen species. So bisphenol A and catechol significantly quenched CL. The strategy provided simple methods for bisphenol A and catechol analysis with a detection limit of 0.07 μM and 0.062 μM, respectively.

**Figure 7:**
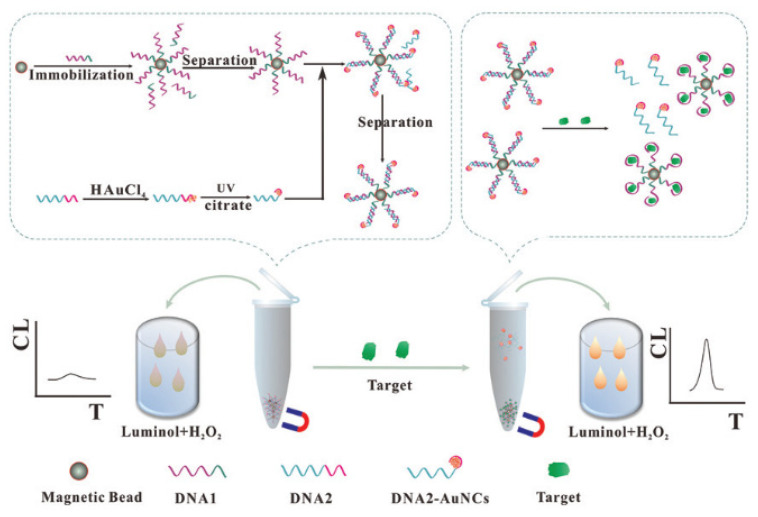
Sensitive detection of kanamycin by the CL sensing strategy based on the catalysis of DNA templated Au NCs. Reprinted from [84] with permission from RSC.

Other template stabilized Au NCs also have catalytic effect on some CL systems. Hemoglobin-stabilized Au NCs displayed a strong catalytic effect on the luminol-NaIO_4_ system [91]. Dopamine can be used as scavenging agents of reactive oxygen species and then inhibited the CL signal. This method was used for the measurement of dopamine in human plasma. Li et al. developed a CL method for detection of kanamycin based on DNA templated Au NCs catalysis and the separation of magnetic beads [84]. As shown in Figure 7, the strategy designed two single-stranded DNAs (DNA1 and DNA2). DNA1 comprised the aptamer of kanamycin. DNA2 consisted of repeat adenosine bases to prepare Au NCs by UV-light-assisted method and a nucleotide sequence to hybridize with DNA1. This strategy provided a versatile method for detection of different targets by altering the aptamer sequence of DNA1.

The catalytic activity of BSA stabilized Ag NCs (BSA/Ag NCs) and BSA stabilized Cu NCs (BSA/Cu NCs) in luminol-H_2_O_2_ system has also been reported [82,85]. Fan et al. firstly prepared four kinds of BSA/Ag NCs using different amount of BSA. BSA/Ag NCs with less BSA had higher affinity to H_2_O_2_ and were beneficial to the generation of ^•^OH and ^1^O_2_ [82]. The determination of H_2_O_2_ was allowed. H_2_O_2_ is the oxidative product of uric acid in the presence of the urate oxidase. CL detection of uric acid was realized with a detection limit of 0.75 μM. Using the similar design strategy, Xu et al. developed a sensitive CL method for detection of cholesterol in milk and human serum samples based on the catalytic activity of BSA/Cu NCs [85]. These works can be applied to the detection of other enzyme-coupled catalyzed biologically molecules.

Cysteine capped Cu NCs (Cys/Cu NCs) also strongly enhanced the CL signal of luminol-H_2_O_2_ reaction [86] and diperiodatoargentate (III)-folic acid reaction [88]. Phenylalanine and tryptophan could enhance the sensitivity of Cys/Cu NCs-luminol-H_2_O_2_ system. Nitrite inhibited CL intensity of Cys/Cu NCs-diperiodatoargentate (III)-folic acid system. Benefiting from these phenomena, the sensors were available for detection of phenylalanine and tryptophan in human serum, and folic acid and nitrite in water, pickled vegetable and sausage samples with satisfactory recoveries.

#### 3.1.2. Methods for Improving Catalytic Efficiency of Metal NCs

In order to further improve the catalytic efficiency of metal NCs, four main strategies are proposed so far, such as cationic metal NCs, metal NCs-MOF structure (metal-organic frameworks, MOFs), bimetal core NCs and synergistic effect of nanomaterials. Cationic Au NCs was designed based on ethanediamine modified on the surface of BSA/Au NCs [83]. The cationic BSA/Au NCs showed higher catalytic activity in the luminol-H_2_O_2_ system than did unmodified Au NCs. The principle was based on the generation of ^1^O_2_ on BSA/Au NCs cationic surface and better affinity between luminol and cationic BSA/Au NCs. The cationic BSA/Au NCs were believed as an artificial peroxidase for potential applications.

In order to improve the stability and catalytic efficiency of Cu NCs, Nano-pores of CuMOFs had been exploited as hosts for encapsulating Cu NCs to form Cu NCs@CuMOFs nanocomposite [89]. The nanocomposite enhanced the CL emission of KMnO_4_-rhodamine 6 G reaction by about 48 times, which was greater than bare Cu NCs (just 15 times). Tramadol could enhance the CL emission of KMnO_4_-rhodamine 6 G-Cu NCs@CuMOFs system. This method displayed good analytical performance with linear range from 0.0030 μM to 2.5 μM, with a detection limit of 0.80 nM. They also proposed that synergetic catalytic effects of BSA/Au NCs-graphene quantum dots (GQDs) and BSA/Ag NCs-GQDs enhanced the weak emission of KMnO_4_-rhodamine B CL reaction [31,32]. Cimetidine can decrease the CL intensity of KMnO_4_-rhodamine B-BSA/Au NCs-GQDs when interacting with these catalysts and reducing their activity. Rabeprazole showed a selective enhancing impact on the KMnO_4_-rhodamine B-BSA/Ag NCs-GQDs CL emission. The method was used for the detection of rabeprazole in human urine.

Compared with single metal NCs, bimetal NCs showed superior catalytic activity to CL reactions. Mokhtarzadeh et al. studied the CL enhancement phenomenon of H_2_O_2_-rhodamine B system in the presence of penicillamine stabilized AuCu NCs [30]. The CL intensity was enhanced by about 15, 40 and 45 times in the presence of pure Au NCs, Cu NCs and the mixture of Au NCs and Cu NCs, respectively. Surprisingly, the AuCu NCs improved the CL emission by about 130-fold. The AuCu NCs decomposed H_2_O_2_ to ^•^OH radicals which oxidize rhodamine B. The concentrations of H_2_O_2_, glucose and xanthine can be detected.

### 3.2. Metal NCs as CL Luminophores

Compared with reports of the catalytic action of metal NCs, the investigation of metal NCs as luminophores of CL is rare. Li et al. designed a CL resonance energy transfer platform for sensitive and label-free detection of trypsin [35]. Bis(2,4,6-trichlorophenyl) oxalate (TCPO)-H_2_O_2_ acted as energy donor and BSA/Au NCs acted as energy acceptor. The BSA/Au NCs produced intense CL by accepting the energy from TCPO-H_2_O_2_ CL reaction. The CL intensity of BSA/Au NCs decreased when BSA was cleft by trypsin. As a consequence, a sensitive CL method for detection of trypsin was accomplished with a wide linear range from 0.01 μg mL^−1^ to 50.0 μg mL^−1^ with a detection limit of 9 ng mL^−1^. They continued to research direct CL of BSA/Au NCs with classic oxidants, such as KMnO_4_, N-bromosuccinimide, K_3_Fe(CN)_6_, H_2_O_2_ and Ce(IV) [34]. The highest CL signal was from acidic KMnO_4_-BSA/Au NCs CL reaction. The possible luminophore was the excited state Mn(II)^*^, originating from the reduction of KMnO_4_ with BSA/Au NCs. H_2_O_2_ can decreased the CL signal of acidic KMnO_4_- BSA/Au NCs system. Hence, the novel CL system was developed for the H_2_O_2_ determination with a linear range from 1.0 × 10^−6^ mol L^−1^ to 1.0 × 10^−4^ mol L^−1^. Cys/Cu NCs also played the role of reductant in the KMnO_4_- Cys/Cu NCs CL system [33]. Luminophore was the excited state Mn(II)^*^, originating from the reduction of KMnO_4_ with Cys/Cu NCs. Yuan et al. utilized hyperbranched polyethyleneimine (hPEI) as a template for the synthesis of hPEI/Ag NCs, which emitted CL by reacting with hydroxy radicals [36]. The emission originated from excited hPEI/Ag NCs. The molecular weight of hPEI affected the CL signal of hPEI/Ag NCs and 10 K hPEI/Ag NCs displayed the strongest CL signal. Tea polyphenols served as antioxidants consumed hydroxy radicals and resulted in a decrease in the CL signal. A sensitive detection method for tea polyphenols was developed based on this phenomenon. The excited state of Cys/Cu NCs also acted as a luminophore in the Ce(IV)-Cu NCs CL system [33].

### 3.3. Metal NCs as CL Quenchers

There are few reports on quenching CL of metal NCs in recent five years. Vahid et al. found that CdSe quantum dots (CdSe QDs) increased the CL intensity of H_2_O_2_-HCO_3_^−^ system based on the CL resonance energy transfer (CRET) between the CL emitters and CdSe QDs and the catalytic activity of CdSe QDs [37]. BSA/Au NCs could prohibit the CRET system and turn off the CL emission. The CL signal was recovered because of the leaching effect of cyanide on Au NCs. This strategy resulted in a highly sensitive and reliable measurement of cyanide in environmental waters and biological samples.

## 4. Summary and Future Perspectives

In the past five years, tremendous literatures on the applications of metal NCs have been reported. In this presented review, we introduce recent research progress in the ECL and CL of metal NCs. Metal NCs with novel catalytic, electrical and optical properties can be obtained on various thiols, protein, DNA, polymer through chemical and electrochemical methods simply, rapidly and cheaply. On account of the advantages of metal NCs, such as small size, good biocompatibility, and luminosity, metal NCs gradually become a multipurpose tool promising for applications in sensing of DNAs and RNAs, metal ions, proteins and enzymes, small biomolecules and so on. This review focuses on the roles of metal NCs in CL and ECL fields, such as luminophores, catalysts, and quenchers. Many efforts have been devoted to enhance the ECL efficiency of metal NCs and the catalytic efficiency of metal NCs in CL system. Some recently reported detection methods using metal NCs were listed in the Table 2.

However, a great deal of challenges and difficulties remain in this exciting field of science. First, the chemical synthesis of metal NCs produces atomically precise but polydisperse clusters. In addition, the stability and dispersion of metal NCs has a certain influence on the luminous efficiency and catalytic efficiency. Therefore, it is urgent to explore new synthesis and purification methods to get metal NCs with single size, good stability and dispersion. Both Cu NCs and Ag NCs can improve their dispersion and control the size by designing DNA structures. DNA synthesis of Au NCs and other metal NCs has not been reported. Protein is one of the main protectants for the synthesis of metal NCs. Can peptides replace proteins in the synthesis of metal NCs? Can the size, dispersion and stability of metal NCs be improved if nanomaterials with special structures are introduced, such as MOF materials? These are all questions worth studying.

Second, although many kinds of metal NCs have been studied, such as Au, Ag, Pt, Cu, Ni, and some alloy NCs. It is still one of the directions to develop other non-noble metal NCs, alloy NCs or doped NCs to reduce costs and develop new properties. At present, the metal cores of alloy nanoclusters are mainly composed of two kinds of metals. There have been no clusters of three or more metallic elements. Doped elements are also very limited, electron-rich element doping may be one direction.

Third, the principles of metal NCs as luminescent agents, catalysts or quenchers are very complex. It is very important to study systematically the influence of metal nucleus, ligand, structure, solvent and charge on the properties of metal NCs. For example, whether the valence state-dependent ECL properties of the Au NCs can also be applied to other metal NCs? What about intramolecular electron transfer and aggregation-induced ECL? Only when one principle or strategy is verified on a variety of metal nanoclusters, its rationality can be effectively explained.

It is expected that with the recognition of the advantages of metal NCs by more researchers, emerging excellent work will be reported in the near future.

## Figures and Tables

**Table 1:** Methods to improve ECL efficiency of Au NCs, AgNCs and Cu NCs.

Position	Methods	ECL System of Au NCs	Ref.
Metal core	Reduction of NCs	*N*-acetyl-L-cysteine stabilized Au NCs + K_2_S_2_O_8_	[15]
		GSH stabilized Au NCs + K_2_S_2_O_8_	
	Pre-oxidation of NCs	L-methionine stabilized Au NCs + TEA	[17]
		Dithiothreitol stabilized Au NCs and BSA/Au NCs + TEA	
	Doping Strategy of NCs	Doping of Ag into BSA/Au NCs + TEA	[38]
	Bimetallic NCs	GSH stabilized AuAg NCs + K_2_S_2_O_8_	[40]
		Au_12_Ag_13_ NCs + TPrA	[41]
Ligand	New Ligand	L-methionine stabilized Au NCs + K_2_S_2_O_8_	[19]
		L-methionine stabilized Au NCs + triethanolamine	[53]
	Double Ligand	Thioglycol/glutathione dual ligand-coated Au NCs + TEA	[54]
	Rigid Host-Guest Assemblies	Mixed L-arginine (ARG) and 6-aza-2-thiothymine (ATT) protected Au NCs + TPrA	[18]
External environment	Novel coreactants	LA-stabilized Au NCs / EDTA	[55]
		LA-stabilized Au NCs / HEPES	[56]
	Coreaction accelerator	BSA/AuNCs + K_2_S_2_O_8_, highly-branched Cu_2_O as the coreaction accelerator	[58]
		BSA/AuNCs + TEA, Cu_2_S snowflake as the coreaction accelerator	[59]
		BSA/AuNCs + K_2_S_2_O_8_, thiocholine produced in situ as the coreaction accelerator	[60]
	Intramolecular Electron Transfer: Binary or ternary nanostructure formed by luminophores, coreactants and coreaction accelerators via covalent attachment	Binary nanostructure formed by LA/Au NCs as the luminophore and DEDA as the coreactant	[20]
		Binary nanostructure formed by TA/Au NCs as the luminophore and DPEA as the coreactant	[61]
		Ternary nanostructure formed by BSA/Au NCs as the luminophore, TAEA as the coreactant and Pd@CuO nanomaterial as the coreaction accelerator	[57]
		Binary nanostructure formed by BSA/Au NCs as the luminophore, TiO_2_ nanosheets as the coreaction accelerator with O_2_ as the coreactant.	[46]
		Ternary nanostructure of BSA/Au NCs- as the luminophore, Cu_2_O@Cu nanoparticles as the coreaction accelerator, and DEDA as the coreactant.	
	Aggregation-Induced ECL	ATT/Au NCs + TEA	[43]
		ATP/Au NCs after Ca^2+^ induced aggregation + HEPES	[44]
	Nanostructure-based Enhancement	BSA/Au NCs + K_2_S_2_O_8_, nanoporous NiGd–Ni_2_O_3_–Gd_2_O_3_@Au nanoparticles as sensor platform	[62]
	Synergistic Effect and ECL resonance energy transfer	Au NCs@ GQDs nanocomposite as the luminophore, GQDs as a donor, Au NCs as an acceptor	[63]
	Biological Signal Amplification	Au NCs dual-labeled with hairpin DNA (H1 and H2) + K_2_S_2_O_8_, using HCR signal amplification.	[64]
Position	Methods	ECL system of Ag NCs	Ref.
Metal core	Design DNA structure to improve the stability and uniformity of Ag NCs	Triplex DNA templated Ag NCs + Na_2_S_2_O_8_,	[65]
External environment	Biological signal amplification	DNA/Ag NCs + K_2_S_2_O_8_, DNAzyme-assisted target recycling and HCR multiple amplification strategy	[66]
	Coreaction accelerators	Ag NCs + K_2_S_2_O_8_, Fe_3_O_4_-CeO_2_ nanocomposites as coreaction accelerator	[67]
	Surface plasmon-enhanced ECL	DNA templated Ag NCs as the luminophore and Au NPs as the localized surface plasmon resonance source	[68]
Metal core	Design DNA structure to improve the stability and less collision of Cu NCs	DNA nanocrane/Cu NCs + K_2_S_2_O_8_	[71]
	Recombination	Nanocomposite DTT/Cu NCs / CNNSs + K_2_S_2_O_8_	[72]
	Doping the rich electronic rare earth element into Cu NCs	Eu^3+^ ion doped GSH stabilized Cu NCs	[73]
External environment	Coreaction accelerators and biological signal amplification	dsDNA/Cu NCs + K_2_S_2_O_8_, TiO_2_ as coreaction accelerator, exonuclease III-assisted amplification and HCR	[70]

**Table 2:** Recently reported ECL and CL biosensors using metal NCs.

Detection Technique	Metal NCs or Probe	Target	Linear Range	LOD	Ref.
ECL	Graphene/Au NCs + K_2_S_2_O_8_	H_2_O_2_	4–24 μM	2 μM	[10]
	BSA/Au NCs + K_2_S_2_O_8_	Dopamine	2.5–7.5 μM	-	[11]
	Graphene/multiwall carbon nanotube/Au NCs + K_2_S_2_O_8_	Hydroquinone	1.0–60 μM	0.3 μM	[12]
		Resorcinol	3.0–70 μM	1.0 μM	
		*p*-Cresol	5.0–60 μM	1.7 μM	
		*p*-Chlorophenol	4–70 μM	1.3 μM	
		2-Bromophenol	5–70 μM	1.7 μM	
	DNA/Ag NCs + K_2_S_2_O_8_	MicroRNA-21	100 aM–100 pM	22 aM	[13]
	BSA/Cu NCs + hydrazine	Dopamine	1.0 × 10^−12^–1.0 × 10^−8^ M	3.5 × 10^−13^ M	[14]
	Met/Au NCs + K_2_S_2_O_8_	Dopamine	0.1–4 μM	32 nM	[19]
	BSA/AuAg NCs + triethylamine	Hg^2+^	10 nM–5 μM	5 nM	[38]
	GSH/AuAg NCs + TEA, Ag NPs as a coreaction accelerator	GSH	5–200 μM	0.90 μM	[39]
	GSH/AuAg NCs + K_2_S_2_O_8_	Dopamine	10 nM–1 mM	2.3 nM	[40]
	Adenosine mono-/di-/triphosphate/Au NCs + TEA	Calmodulin	0.3–50 μg mL^−1^	0.1 μg mL^−1^	[44]
	BSA/Au NCs + DEDA	CEA	1 pg mL^−1^–1 ng mL^−1^	0.43 pg mL^−1^	[46]
		MUC1	10 fg mL^−1^–1 ng mL^−1^	5.8 fg mL^−1^	
	BSA/Au NCs + H_2_O_2_	5-Methylcytosine-DNA	0.01–50 nM	3.46 pM	[47]
		Ten-eleven translocation 1 protein	1–10 μg mL^−1^	0.37 μg mL^−1^	
		T4 β-glucosyltransferase	0.5–50 unit mL^−1^	0.176 unit mL^−1^	
	BSA/Au NCs + TEA	Concanavalin A	0.004–90 ng mL^−1^	0.001 ng mL^−1^	[49]
	NAC/Au NCs + K_2_S_2_O_8_	GSH	1.0 × 10^−9^–1.0 × 10^−5^ M and 1.0 × 10^−5^–1.0 × 10^−1^ M	3.2 × 10^−10^ M	[50]
	Met/Au NCs + triethanolamine	α-Fetoprotein	3 fg mL^−1^–0.1 ng mL^−1^	1 fg mL^−1^	[53]
	BSA/Au NCs + TAEA	CEA	100 fg mL^−1^–100 ng mL^−1^	16 fg mL^−1^	[57]
	BSA/Au NCs + K_2_S_2_O_8_	Procalcitonin	10 fg mL^−1^–100 ng mL^−1^	2.90 fg mL^−1^	[58]
	BSA/Au NCs + TEA	Procalcitonin	10 fg mL^−1^–100 ng mL^−1^	2.36 fg mL^−1^	[59]
	BSA/Au NCs + K_2_S_2_O_8_	ATCI	0.50 nM–0.47 mM	0.17 nM	[60]
	TA/Au NCs +DPEA	Mucin 1	1 fg mL^−1^–1 ng mL^−1^	0.54 fg mL^−1^	[61]
	BSA/Au NCs + K_2_S_2_O_8_	CEA	10^−4^–5 ng mL^−1^	0.03 pg mL^−1^	[62]
	BSA/Au NCs/GQDs + TEA	Pentoxifylline	7.0 × 10^−7^–1.2 × 10^−4^ M	9.0 × 10^−8^ M	[63]
	GSH/Au NCs + K_2_S_2_O_8_	Cardiac troponin I	5 fg mL^−1^–50 ng mL^−1^	1.01 fg mL^−1^	[64]
	Triplex DNA/Ag NCs	Cys	0.5–50 μM	0.5 μM	[65]
	DNA/Ag NCs + K_2_S_2_O_8_	Thrombin	10.0 fM–10.0 nM	4.5 fM	[66]
	Ag NCs + K_2_S_2_O_8_	CCND1	50 fg mL^−1^–50 ng mL^−1^	28 fg mL^−1^	[67]
	DNA/AgNCs + K_2_S_2_O_8_	MicroRNA-21	1 aM–10^4^ fM	0.96 aM	[68]
	DNA/Cu NCs + K_2_S_2_O_8_	MicroRNA-21	100 aM–100 pM	19.05 aM	[70]
	DNA/Cu NCs + K_2_S_2_O_8_	MicroRNA-155	100 aM–100 pM	36 aM	[71]
	DTT/Cu NCs + K_2_S_2_O_8_	Hg^2+^	0.5–10 nM	0.01 nM	[72]
	Eu^3+^-Cu NCs	Dopamine	1.0 × 10^−11^–5.0 × 10^−4^ M	1.0 × 10^−11^ M	[73]
	Pt NCs/graphene + TEA	Cu^2+^	1.0 × 10^−4^–2.0 × 10^−1^ mg L^−1^	1.0 × 10^−4^ mg L^−1^	[74]
	Ni NCs + TPrA	Creatinine	5 nM–1 mM	0.5 nM	[75]
	DNA/Ag NCs + K_2_S_2_O_8_	Thrombin	0.001–1000 pM	0.165 fM	[76]
		MicroRNA-21	0–10^3^ pM, 10^−5^–10^3^ pM	4.97 aM	
	GSH/Au NCs + K_2_S_2_O_8_	Cardiac troponin I	50 fg mL^−1^–50 ng mL^−^	9.73 fg mL^−1^	[77]
	g-C_3_N_4_ as a ECL emitter, BSA/Au NCs as catalyst	Protein kinase A	0.02–20 U mL^−1^	0.005 U mL^−1^	[78]
	Pt NCs both as the acceptor and donor	MicroRNA-141	10 aM–100 nM	3.3 aM	[79]
CL	Penicillamine/AuCu NCs	H_2_O_2_	0.2–2000 nM	0.13 nM	[30]
		Glucose	0.1–400 μM	30 nM	
		Xanthine	0.1–200 μM	38 nM	
	KMnO_4_ + rhodamine B enhanced by BSA/Au NCs and GQDs	Cimetidine	0.8–200 ng mL^−1^	0.3 ng mL^−1^	[31]
	KMnO_4_ + rhodamine B enhanced by BSA/Ag NCs and GQDs	Rabeprazole	4–133 ng mL^−1^	1.1 ng mL^−1^	[32]
	Cysteine/Cu NCs + cerium	Trihexyphenidyl hydrochloride	0.1–10.0 μM	49.0 nM	[33]
	BSA/Au NCs + KMnO_4_	H_2_O_2_	1.0 ×10^−6^–1.0 × 10^−4^M	5.0×10^−7^ M	[34]
	BSA/AuNCs as an energy acceptor and bis(2,4,6-tri-chlorophenyl)- oxalate–hydrogen peroxide as an energy donor	Trypsin	0.01–50.0 μg mL^−1^	9 ng mL^−1^	[35]
	hPEI-AgNCs + H_2_O_2_	Tea polyphenols	2.52–76.2 μM	2.52 μM	[36]
	H_2_O_2_-HCO_3_^−^ system, CdSe QDs as catalyst, BSA/Au NCs as quencher	Cyanide	2–225 nM	0.46 nM	[37]
	Au NCs as catalyst	Nitrite	5 μM–0.1 mM	4.7 μM	[80]
	Au NCs as catalyst	Catechol	0.1–10 μM	0.062 μM	[81]
	Ag NCs as catalyst	H_2_O_2_	0.14–100 μM	0.016 μM	[82]
		Uric acid	2–100 μM	0.75 μM	
	Au NCs as catalyst	Kanamycin	0.2–4.4 nM	0.035 nM	[84]
	Cu NCs as catalyst	Cholesterol	0.05–10 mM	1.5 μM	[85]
	Cu NCs as catalyst	Phenylalanine	1.0 × 10^−6^–2.7 × 10^−5^ M	8.4 × 10^−7^ M	[86]
		Tryptophan	1.0 × 10^−^^7^–3.0 × 10^−^^5^ M	7.5 × 10^−^^8^ M	
	Cu NCs as catalyst	Tryptophan	2.0 × 10^−^^7^–10^−4^ M	6 × 10^−8^ M	[87]
	Cu NCs as catalyst	Nitrite	1.0–80.0 μM	0.0954 μM	[88]
		Folic acid	0.1–10.0 μM	3.0 μM	
	Cu NCs@CuMOF as catalyst	Tramadol	0.0030–2.5 μM	0.80 nM	[89]
	BSA/ZnCu NCs as catalyst	H_2_O_2_	5.0 × 10^−^^9^–1.0 × 10^−6^ M	3.0 × 10^−10^ M	[90]
	Hemoglobin/Au NCs as catalyst	Dopamine	0.3–9.0 nM	0.1 nM	[91]
	Au NCs as catalyst	Bisphenol A	2.0 × 10^−^^7^–1.0 × 10^−5^ M	1.0 × 10^−6^ M	[92]

## References

[1] Halawa M.I., Li B.S., Xu G. (2020). Novel Synthesis of Thiolated Gold Nanoclusters Induced by Lanthanides for Ultrasensitive and Luminescent Detection of the Potential Anthrax Spores’ Biomarker. ACS Appl. Mater. Interfaces.

[2] Wang J., Lin X., Shu T., Su L., Liang F., Zhang X. (2019). Self-Assembly of Metal Nanoclusters for Aggregation-Induced Emission. Int. J. Mol. Sci..

[3] Xu J., Shang L. (2018). Emerging applications of near-infrared fluorescent metal nanoclusters for biological imaging. Chin. Chem. Lett..

[4] Halawa M.I., Wu F., Nsabimana A., Lou B., Xu G. (2018). Inositol directed facile “green” synthesis of fluorescent gold nanoclusters as selective and sensitive detecting probes of ferric ions. Sens. Actuators B Chem..

[5] Halawa M.I., Wu F., Fereja T.H., Lou B., Xu G. (2018). One-pot green synthesis of supramolecular beta-cyclodextrin functionalized gold nanoclusters and their application for highly selective and sensitive fluorescent detection of dopamine. Sens. Actuators B Chem..

[6] Halawa M.I., Lai J., Xu G. (2018). Gold nanoclusters: Synthetic strategies and recent advances in fluorescent sensing. Mater. Today Nano.

[7] Halawa M.I., Gao W., Saqib M., Kitte S.A., Wu F., Xu G. (2017). Sensitive detection of alkaline phosphatase by switching on gold nanoclusters fluorescence quenched by pyridoxal phosphate. Biosens. Bioelectron..

[8] Díez I., Pusa M., Kulmala S., Jiang H., Walther A., Goldmann A.S., Müller A.H.E., Ikkala O., Ras R.H.A. (2009). Color Tunability and Electrochemiluminescence of Silver Nanoclusters. Angew. Chem. Int. Ed..

[9] Fang Y.-M., Song J., Li J., Wang M., Yang H., Sun J.-J., Chen G.-N. (2011). Electrogenerated chemiluminescence from Au nanoclusters. Chem. Commun..

[10] Chen Y., Shen Y., Sun D., Zhang H., Tian D., Zhang J., Zhu J.-J. (2011). Fabrication of a dispersible graphene/gold nanoclusters hybrid and its potential application in electrogenerated chemiluminescence. Chem. Commun..

[11] Li L., Liu H., Shen Y., Zhang J., Zhu J.J. (2011). Electrogenerated Chemiluminescence of Au Nanoclusters for the Detection of Dopamine. Anal. Chem..

[12] Yuan D., Chen S., Yuan R., Zhang J., Zhang W. (2013). An electrogenerated chemiluminescence sensor prepared with a graphene/multiwall carbon nanotube/gold nanocluster hybrid for the determination of phenolic compounds. Analyst.

[13] Chen A.-Y., Ma S., Zhuo Y., Chai Y., Yuan R. (2016). In Situ Electrochemical Generation of Electrochemiluminescent Silver Naonoclusters on Target-Cycling Synchronized Rolling Circle Amplification Platform for MicroRNA Detection. Anal. Chem..

[14] Zhao M., Chen A.-Y., Huang D., Zhuo Y., Chai Y.-Q., Yuan R. (2016). Cu Nanoclusters: Novel Electrochemiluminescence Emitters for Bioanalysis. Anal. Chem..

[15] Peng H., Jian M., Deng H., Wang W., Huang Z., Huang K., Liu A., Chen W. (2017). Valence States Effect on Electrogenerated Chemiluminescence of Gold Nanocluster. ACS Appl. Mater. Interfaces.

[16] Peng H., Huang Z., Wu W., Liu M., Huang K., Yang Y., Deng H., Xia X.-H., Chen W. (2019). Versatile High-Performance Electrochemiluminescence ELISA Platform Based on a Gold Nanocluster Probe. ACS Appl. Mater. Interfaces.

[17] Peng H., Huang Z., Sheng Y., Zhang X., Deng H., Chen W., Liu J. (2019). Pre-oxidation of Gold Nanoclusters Results in a 66% Anodic Electrochemiluminescence Yield and Drives Mechanistic Insights. Angew. Chem. Int. Ed..

[18] Yang L., Zhang B., Fu L., Fu K., Zou G. (2019). Efficient and Monochromatic Electrochemiluminescence of Aqueous-Soluble Au Nanoclusters via Host–Guest Recognition. Angew. Chem. Int. Ed..

[19] Peng H., Deng H., Jian M., Liu A., Bai F., Lin X., Chen W. (2016). Electrochemiluminescence sensor based on methionine-modified gold nanoclusters for highly sensitive determination of dopamine released by cells. Microchim. Acta.

[20] Wang T., Wang G., Padelford J.W., Jiang J., Wang G. (2016). Near-Infrared Electrogenerated Chemiluminescence from Aqueous Soluble Lipoic Acid Au Nanoclusters. J. Am. Chem. Soc..

[21] Guo Y., Pan X., Zhang W., Hu Z., Wong K.-W., He Z., Li H.-W. (2020). Label-free probes using DNA-templated silver nanoclusters as versatile reporters. Biosens. Bioelectron..

[22] Li S., Liu Y., Ma Q. (2019). Nanoparticle-based electrochemiluminescence cytosensors for single cell level detection. TrAC Trends Anal. Chem..

[23] He S., Ding Z. (2018). Progress in electrochemistry and electrochemiluminescence of metal clusters. Curr. Opin. Electrochem..

[24] Rizwan M., Mohd-Naim N., Ahmed M.U. (2018). Trends and Advances in Electrochemiluminescence Nanobiosensors. Sensors.

[25] Jiang H., Wang X. (2017). Progress of Metal Nanoclusters-based Electrochemiluminescent Analysis. Chin. J. Anal. Chem..

[26] Zhai Q., Li J., Wang E. (2017). Recent Advances Based on Nanomaterials as Electrochemiluminescence Probes for the Fabrication of Sensors. Chem. Electro.Chem..

[27] Yu X., Wang Q. (2011). The determination of copper ions based on sensitized chemiluminescence of silver nanoclusters. Microchim. Acta.

[28] Hu L., Yuan Y., Zhang L., Zhao J., Majeed S., Xu G. (2013). Copper nanoclusters as peroxidase mimetics and their applications to H_2_O_2_ and glucose detection. Anal. Chim. Acta.

[29] Wang X.-X., Wu Q., Shan Z., Huang Q.-M. (2011). BSA-stabilized Au clusters as peroxidase mimetics for use in xanthine detection. Biosens. Bioelectron..

[30] Mokhtarzadeh E., Abolhasani J., Hassanzadeh J., Elham M., Jafar A., Javad H. (2019). Rhodamine B Chemiluminescence Improved by Mimetic AuCu Alloy Nanoclusters and Ultrasensitive Measurement of H_2_O_2_, Glucose and Xanthine. Anal. Sci..

[31] Yousefzadeh A., Abolhasani J., Hassanzadeh J., Somi M.H. (2019). Ultrasensitive chemiluminescence assay for cimetidine detection based on the synergistic improving effect of Au nanoclusters and graphene quantum dots. Luminescence.

[32] Yousefzadeh A., Abolhasani J., Hassanzadeh J., Somi M.H. (2019). A Highly Efficient Chemiluminescence System Based on an Enhancing Effect of Ag Nanoclusters/Graphene Quantum Dots Mixture for Ultrasensitive Detection of Rabeprazole. Anal. Sci..

[33] Chen X., Zhang J., Li Y., Han S. (2018). Chemiluminescence of copper nanoclusters and its application for trihexyphenidyl hydrochloride detection. Luminescence.

[34] You X., Li Y. (2019). Direct chemiluminescence of fluorescent gold nanoclusters with classic oxidants for hydrogen peroxide sensing. Arab. J. Chem..

[35] You X., Li Y., Li B., Ma J. (2016). Gold nanoclusters-based chemiluminescence resonance energy transfer method for sensitive and label-free detection of trypsin. Talanta.

[36] Zhao S., Chen C., Li Z., Yuan Z., Lu C. (2017). Hydroxyl radical induced chemiluminescence of hyperbranched polyethyleneimine protected silver nanoclusters and its application in tea polyphenols detection. Anal. Methods.

[37] Vahid B., Hassanzadeh J., Khodakarami B. (2019). CdSe quantum dots-sensitized chemiluminescence system and quenching effect of gold nanoclusters for cyanide detection. Spectrochim. Acta Part A Mol. Biomol. Spectrosc..

[38] Zhai Q., Xing H., Zhang X., Li J., Wang E. (2017). Enhanced Electrochemiluminescence Behavior of Gold–Silver Bimetallic Nanoclusters and Its Sensing Application for Mercury(II). Anal. Chem..

[39] Yang F., Jiang X.-Y., Liang W.-B., Chai Y.-Q., Yuan R., Zhuo Y. (2020). 3D Matrix-Arranged AuAg Nanoclusters As Electrochemiluminescence Emitters for Click Chemistry-Driven Signal Switch Bioanalysis. Anal. Chem..

[40] Tang Y., Xu J., Xiong C., Xiao Y., Zhang X., Wang S. (2019). Enhanced electrochemiluminescence of gold nanoclusters via silver doping and their application for ultrasensitive detection of dopamine. Analyst.

[41] Chen S., Ma H., Padelford J.W., Qinchen W., Yu W., Wang S., Kang X., Wang G. (2019). Near Infrared Electrochemiluminescence of Rod-Shape 25-Atom AuAg Nanoclusters That Is Hundreds-Fold Stronger Than That of Ru(bpy)_3_ Standard. J. Am. Chem. Soc..

[42] Goswami N., Yao Q., Luo Z., Li J., Chen T., Xie J. (2016). Luminescent Metal Nanoclusters with Aggregation-Induced Emission. J. Phys. Chem. Lett..

[43] Peng H., Huang Z., Deng H., Wu W., Huang K., Li Z., Chen W., Liu J. (2019). Dual Enhancement of Gold Nanocluster Electrochemiluminescence: Electrocatalytic Excitation and Aggregation-Induced Emission. Angew. Chem. Int. Ed..

[44] Jiang H., Qin Z., Zheng Y., Liu L., Wang X. (2019). Aggregation-Induced Electrochemiluminescence by Metal-Binding Protein Responsive Hydrogel Scaffolds. Small.

[45] Han S., Zhang Z., Li S., Qi L., Xu G. (2016). Chemiluminescence and electrochemiluminescence applications of metal nanoclusters. Sci. China Ser. B Chem..

[46] Zhou Y., Chai Y.-Q., Yuan R. (2019). Highly Efficient Dual-Polar Electrochemiluminescence from Au_25_ Nanoclusters: The Next Generation of Multibiomarker Detection in a Single Step. Anal. Chem..

[47] Jiang W., Yin H., Zhou Y., Duan J., Li H., Wang M., Waterhouse G.I.N., Ai S. (2018). A novel electrochemiluminescence biosensor for the detection of 5-methylcytosine, TET 1 protein and beta-glucosyltransferase activities based on gold nanoclusters-H_2_O_2_ system. Sens. Actuators B Chem..

[48] Li W.J., Wang X., Jiang T., Ma X., Tian H. (2020). One-pot synthesis of beta-cyclodextrin modified Au nanoclusters with near-infrared emission. Chem. Commun..

[49] Chen S., Fan Y., Zhang C., He Y., Wei S. (2017). Quenched solid-state electrochemiluminescence of gold nanoclusters and the application in the ultrasensitive detection of concanavalin A. Electrochim. Acta.

[50] Peng H.-P., Jian M.-L., Huang Z.-N., Wang W.-J., Deng H.-H., Wu W.-H., Liu A.-L., Xia X.-H., Chen W. (2018). Facile electrochemiluminescence sensing platform based on high-quantum-yield gold nanocluster probe for ultrasensitive glutathione detection. Biosens. Bioelectron..

[51] Kim J.M., Jeong S., Song J.K., Kim J. (2018). Near-infrared electrochemiluminescence from orange fluorescent Au nanoclusters in water. Chem. Commun..

[52] Kang Y., Kim J. (2020). Electrochemiluminescence of Glutathione-Stabilized Au Nanoclusters Fractionated by Gel Electrophoresis in Water. ChemElectroChem.

[53] Yu L., Zhang Q., Kang Q., Zhang B., Shen D., Zou G. (2020). Near-Infrared Electrochemiluminescence Immunoassay with Biocompatible Au Nanoclusters as Tags. Anal. Chem..

[54] Jiang H., Liu L., Wang X. (2017). Red-emitted electrochemiluminescence by yellow fluorescent thioglycol/glutathione dual thiolate co-coated Au nanoclusters. Nanoscale.

[55] Wang T., Padelford J.W., Ma H., Gubitosi-Raspino M.F., Wang G. (2017). Near-Infrared Electrochemiluminescence from Au Nanoclusters Enhanced by EDTA and Modulated by Ions. ChemElectroChem.

[56] Wang T., Ma H., Padelford J.W., Lobo E., Tran M.T., Zhao F., Fang N., Wang G. (2018). Metal ions-modulated near-infrared electrochemiluminescence from Au nanoclusters enhanced by 4-(2-Hydroxyethyl)-1-piperazineethanesulfonic acid at physiological pH. Electrochim. Acta.

[57] Zhou Y., Chen S., Luo X., Chai Y., Yuan R. (2018). Ternary Electrochemiluminescence Nanostructure of Au Nanoclusters as a Highly Efficient Signal Label for Ultrasensitive Detection of Cancer Biomarkers. Anal. Chem..

[58] Jia Y., Yang L., Xue J., Ren X., Zhang N., Fan D., Wei Q., Ma H. (2019). Highly-branched Cu_2_O as well-ordered co-reaction accelerator for amplifying electrochemiluminescence response of gold nanoclusters and procalcitonin analysis based on protein bioactivity maintenance. Biosens. Bioelectron..

[59] Jia Y., Yang L., Xue J., Zhang N., Fan D., Ma H., Ren X., Hu L., Wei Q. (2019). Bioactivity-Protected Electrochemiluminescence Biosensor Using Gold Nanoclusters as the Low-Potential Luminophor and Cu_2_S Snowflake as Co-reaction Accelerator for Procalcitonin Analysis. ACS Sens..

[60] Zhang C., Fan Y., Zhang H., Chen S., Yuan R. (2018). An ultrasensitive signal-on electrochemiluminescence biosensor based on Au nanoclusters for detecting acetylthiocholine. Anal. Bioanal. Chem..

[61] Yang F., Zhong X., Jiang X., Zhuo Y., Yuan R., Wei S. (2019). An ultrasensitive aptasensor based on self-enhanced Au nanoclusters as highly efficient electrochemiluminescence indicator and multi-site landing DNA walker as signal amplification. Biosens. Bioelectron..

[62] Lv X., Ma H., Wu D., Yan L., Ji L., Liu Y., Pang X., Du B., Wei Q. (2016). Novel gold nanocluster electrochemiluminescence immunosensors based on nanoporous NiGd–Ni_2_O_3_–Gd_2_O_3_ alloys. Biosens. Bioelectron..

[63] Zhang X.-L., Li X., Li X.-T., Gao Y., Feng F., Yang G. (2019). Electrochemiluminescence sensor for pentoxifylline detection using Au nanoclusters@graphene quantum dots as an amplified electrochemiluminescence luminophore. Sens. Actuators B Chem..

[64] Zhu L., Ye J., Yan M., Zhu Q., Wang S., Huang J., Sun J. (2019). Electrochemiluminescence Immunosensor Based on Au Nanocluster and Hybridization Chain Reaction Signal Amplification for Ultrasensitive Detection of Cardiac Troponin I. ACS Sens..

[65] Feng L., Wu L., Xing F., Hu L., Ren J., Qu X. (2017). Novel electrochemiluminescence of silver nanoclusters fabricated on triplex DNA scaffolds for label-free detection of biothiols. Biosens. Bioelectron..

[66] Jie G., Tan L., Zhao Y., Wang X. (2017). A novel silver nanocluster in situ synthesized as versatile probe for electrochemiluminescence and electrochemical detection of thrombin by multiple signal amplification strategy. Biosens. Bioelectron..

[67] Zhou Y., Chen M., Zhuo Y., Chai Y., Xu W., Yuan R. (2017). In Situ Electrodeposited Synthesis of Electrochemiluminescent Ag Nanoclusters as Signal Probe for Ultrasensitive Detection of Cyclin-D1 from Cancer Cells. Anal. Chem..

[68] Feng X., Han T., Xiong Y., Wang S., Dai T., Chen J., Zhang X., Wang G. (2019). Plasmon-Enhanced Electrochemiluminescence of Silver Nanoclusters for microRNA Detection. ACS Sens..

[69] Song Q., Shi Y., He D., Xu S., Ouyang J. (2014). Sequence-Dependent dsDNA-Templated Formation of Fluorescent Copper Nanoparticles. Chem. Eur. J..

[70] Liao H., Zhou Y., Chai Y.-Q., Yuan R. (2018). An ultrasensitive electrochemiluminescence biosensor for detection of MicroRNA by in-situ electrochemically generated copper nanoclusters as luminophore and TiO_2_ as coreaction accelerator. Biosens. Bioelectron..

[71] Zhou Y., Wang H., Zhang H., Chai Y.-Q., Yuan R. (2018). Programmable Modulation of Copper Nanoclusters Electrochemiluminescence via DNA Nanocranes for Ultrasensitive Detection of microRNA. Anal. Chem..

[72] Liu H., Gao X., Zhuang X., Tian C., Wang Z., Li Y., Rogach A.L. (2019). A specific electrochemiluminescence sensor for selective and ultra-sensitive mercury(II) detection based on dithiothreitol functionalized copper nanocluster/carbon nitride nanocomposites. Analyst.

[73] Zhuang X., Gao X., Tian C., Cui D.-L., Luan F., Wang Z., Xiong Y., Chen L. (2020). Synthesis of europium(iii)-doped copper nanoclusters for electrochemiluminescence bioanalysis. Chem. Commun..

[74] Yang Y., Wu W., Wang Q., Xiao H., Kuang Y., Liu C. (2016). Novel anodic electrochemiluminescence system of Pt nanocluster/graphene hybrids for ultrasensitive detection of Cu^2^^+^. J. Electroanal. Chem..

[75] Babamiri B., Salimi A., Hallaj R., Hasanzadeh M. (2018). Nickel nanoclusters as a novel emitter for molecularly imprinted electrochemiluminescence based sensor toward nanomolar detection of creatinine. Biosens. Bioelectron..

[76] Ge J., Li C., Zhao Y., Yu X., Jie G. (2019). Versatile “on–off” biosensing of thrombin and miRNA based on Ag(i) ion-enhanced or Ag nanocluster-quenched electrochemiluminescence coupled with hybridization chain reaction amplification. Chem. Commun..

[77] Zhu H., Ye J., Yan M., Zhu Q., Zhu H. (2019). A wavelength-resolved electrochemiluminescence resonance energy transfer ratiometric immunosensor for detection of cardiac troponin I. Analyst.

[78] Luo Q.-X., Li Y., Liang R.-P., Cao S.-P., Jin H.-J., Qiu J.-D. (2020). Gold nanoclusters enhanced electrochemiluminescence of g-C_3_N_4_ for protein kinase activity analysis and inhibition. J. Electroanal. Chem..

[79] Wang C., Chen M., Han Q., Wu J., Zhao X., Fu Y. (2020). A three-dimensional DNA nanomachine with target recycling amplification technology and multiple electrochemiluminescence resonance energy transfer for sensitive microRNA-141 detection. Biosens. Bioelectron..

[80] Sui Y., Deng M., Xu S., Chen F. (2015). Gold nanocluster-enhanced peroxynitrous acid chemiluminescence for high selectivity sensing of nitrite. RSC Adv..

[81] Yang D., He Y., Sui Y., Chen F. (2017). Determination of catechol in water based on gold nanoclusters-catalyzed chemiluminescence. J. Luminiscence.

[82] Sheng Y., Yang H., Wang Y., Han L., Zhao Y., Fan A. (2017). Silver nanoclusters-catalyzed luminol chemiluminescence for hydrogen peroxide and uric acid detection. Talanta.

[83] Han L., Li Y., Fan A. (2018). Improvement of mimetic peroxidase activity of gold nanoclusters on the luminol chemiluminescence reaction by surface modification with ethanediamine. Luminiscence.

[84] Yao Y., Wang X., Duan W., Li F. (2018). A label-free, versatile and low-background chemiluminescence aptasensing strategy based on gold nanocluster catalysis combined with the separation of magnetic beads. Analyst.

[85] Xu S., Wang Y., Zhou D., Kuang M., Fang D., Yang W., Wei S., Ma L. (2016). A novel chemiluminescence sensor for sensitive detection of cholesterol based on the peroxidase-like activity of copper nanoclusters. Sci. Rep..

[86] Borghei Y.-S., Hosseini M., Khoobi M., Ganjali M.R. (2017). Copper nanocluster-enhanced luminol chemiluminescence for high-selectivity sensing of tryptophan and phenylalanine. Luminescence.

[87] Wu X., Hu X., Wang G. (2016). Enhanced chemiluminescence of the luminol-hydrogen peroxide system by copper nanoclusters and its analytical application. Chem. Res. Appl..

[88] Han S., Chen X. (2019). Copper nanoclusters-enhanced chemiluminescence for folic acid and nitrite detection. Spectrochim. Acta Part A Mol. Biomol. Spectrosc..

[89] Yousefzadeh A., Hassanzadeh J., Mousavi S.M.J., Yousefzadeh M. (2019). Surface molecular imprinting and powerfully enhanced chemiluminescence emission by Cu nanoclusters/MOF composite for detection of tramadol. Sens. Actuators B Chem..

[90] Chen H., Lin L., Li H., Li J., Lin J.-M. (2015). Aggregation-Induced Structure Transition of Protein-Stabilized Zinc/Copper Nanoclusters for Amplified Chemiluminescence. ACS Nano.

[91] Li Y., Peng W., You X. (2017). Determination of dopamine by exploiting the catalytic effect of hemoglobin–stabilized gold nanoclusters on the luminol–NaIO4 chemiluminescence system. Microchim. Acta.

[92] Yang N., He Y., Sui Y., Chen F. (2016). Gold nanoclusters-catalyzed rhodamine 6G–K_3_Fe(CN)_6_ chemiluminescence and its application. Anal. Methods.

